# Supplementary material for: Estimating the effects of temperature on transmission of the human malaria parasite, Plasmodium falciparum
Source: Nat Commun. 2024 Apr 22;15:3230. doi: 10.1038/s41467-024-47265-w (PMC11035611; doi:10.1038/s41467-024-47265-w)
Supplement: Supplementary file 1 — Supplementary Information [file 41467_2024_47265_MOESM1_ESM.docx]

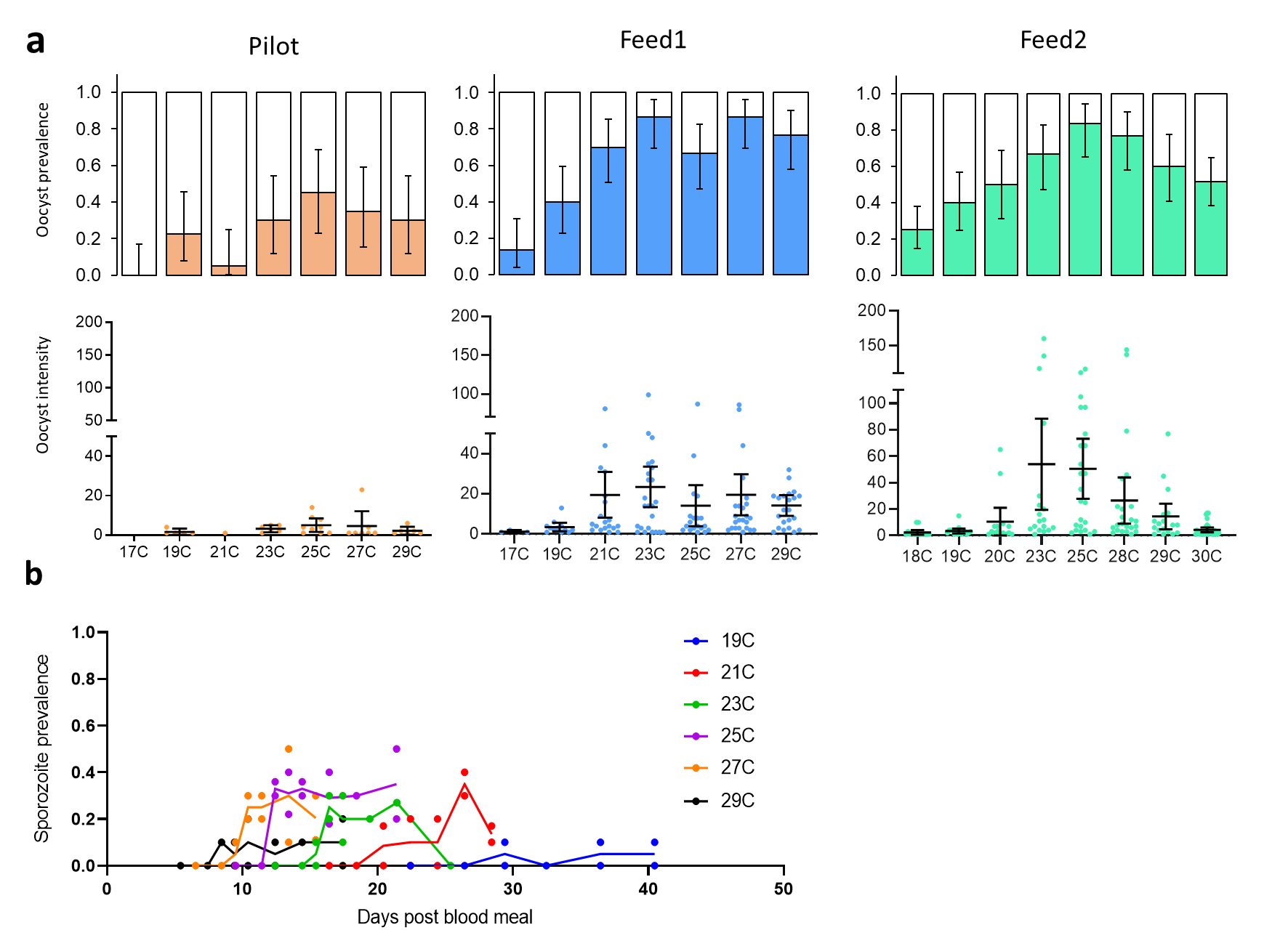


**Supplementary Figure 1. Oocyst infection prevalence and mean intensity in pilot study followed by two main experimental feeds, and resulting sporozoite prevalence for pilot study.** Error bars indicate 95% confidence intervals (Clopper-Pearson 95% confidence intervals for prevalence data). Individual dots in (a) indicate oocyst count in individual mosquito midguts infected with any oocysts (n=199, 210 and 310 mosquitoes dissected for Pilot, Feed1 and Feed2, respectively). Individual dots and solid lines in (b) indicate sporozoite prevalence for each mosquito container and the linear interpolation of mean prevalence of these dots, respectively. Sporozoite data for Feed1 and Feed2 are presented in Fig. 1.


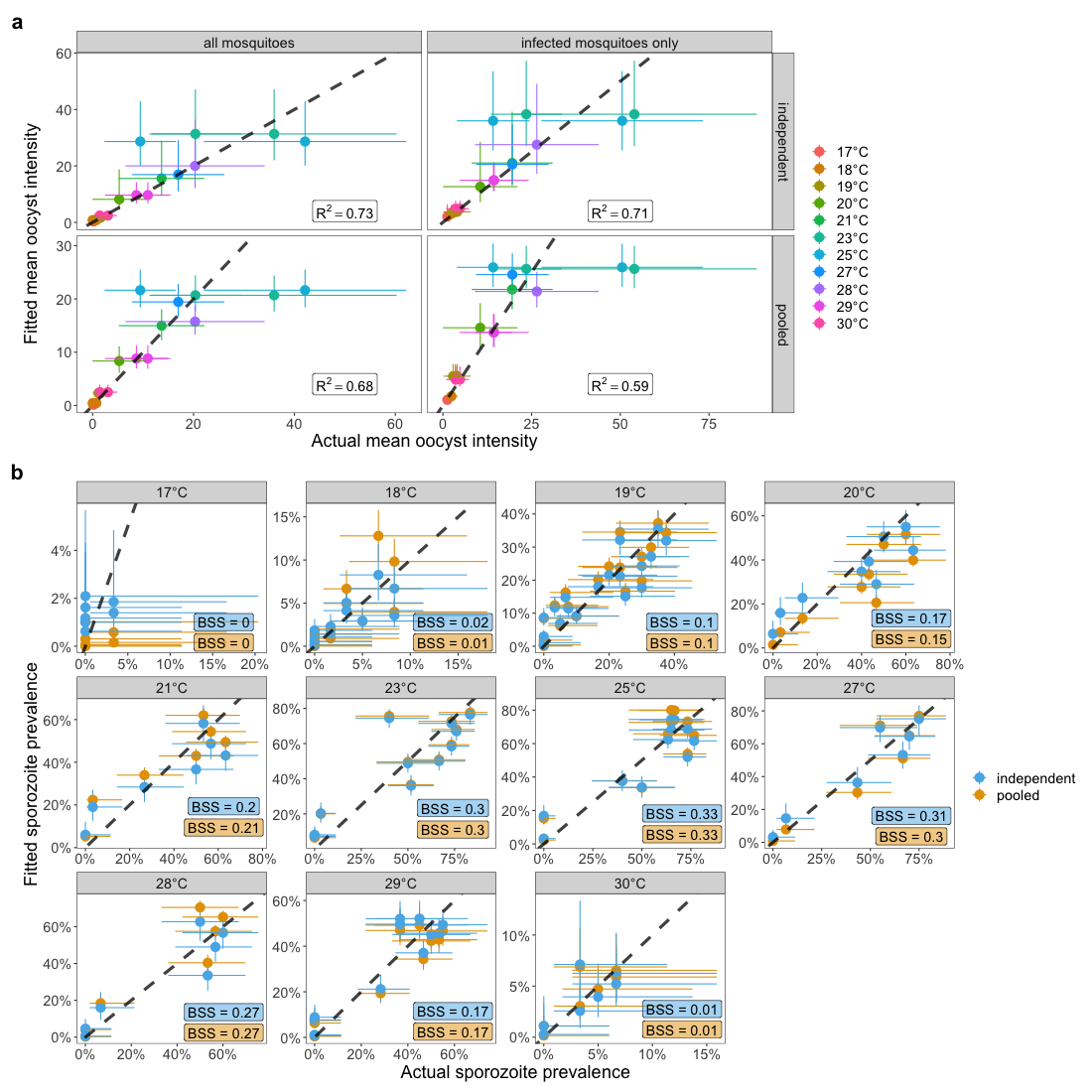


Supplementary Figure 2. Actual vs fitted sporozoite prevalence and mean oocyst intensity. Uncertainty in the data is shown by the 95% confidence intervals for sporozoite prevalence (n=4195) or mean oocyst count (n=520). Uncertainty in the model estimates is shown by the 95% credible intervals (2.5% – 97.5% quantiles of the MCMC posterior samples). y=x is shown by dashed-black line. To assess model performance, we calculated the coefficient of determination (R^2^) for the mean oocyst intensity point estimates and the Brier skill scores (BSS) for the presence of sporozoites in individual mosquitoes. In all cases for the model estimates the posterior median values were used.


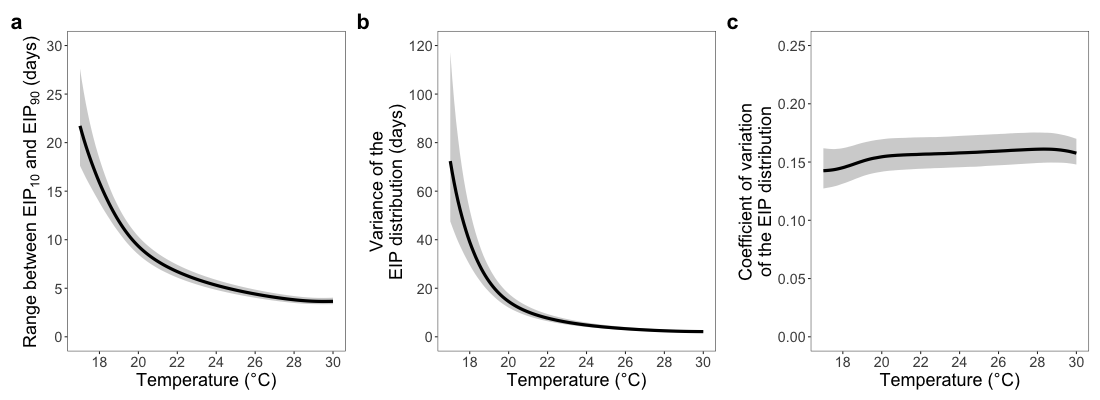


Supplementary Figure 3. Increases in temperature decrease the estimated dispersion, but not normalised dispersion, of the extrinsic incubation period. For all plots, uncertainty is shown by the 95% credible intervals and the solid line shows the median posterior value.

**Supplementary** **Figure 4. First sporozoite infection observed in the current (Feed1 and Feed2) and Nikolaev’s original study^1^.** The pooled model estimates of EIP_10_ (equivalent to first sporozoite infection) and EIP_50_ (median EIP) from our model are compared with empirical data (95% credible intervals were removed for better visibility of the data) and the Detinova degree-day model.


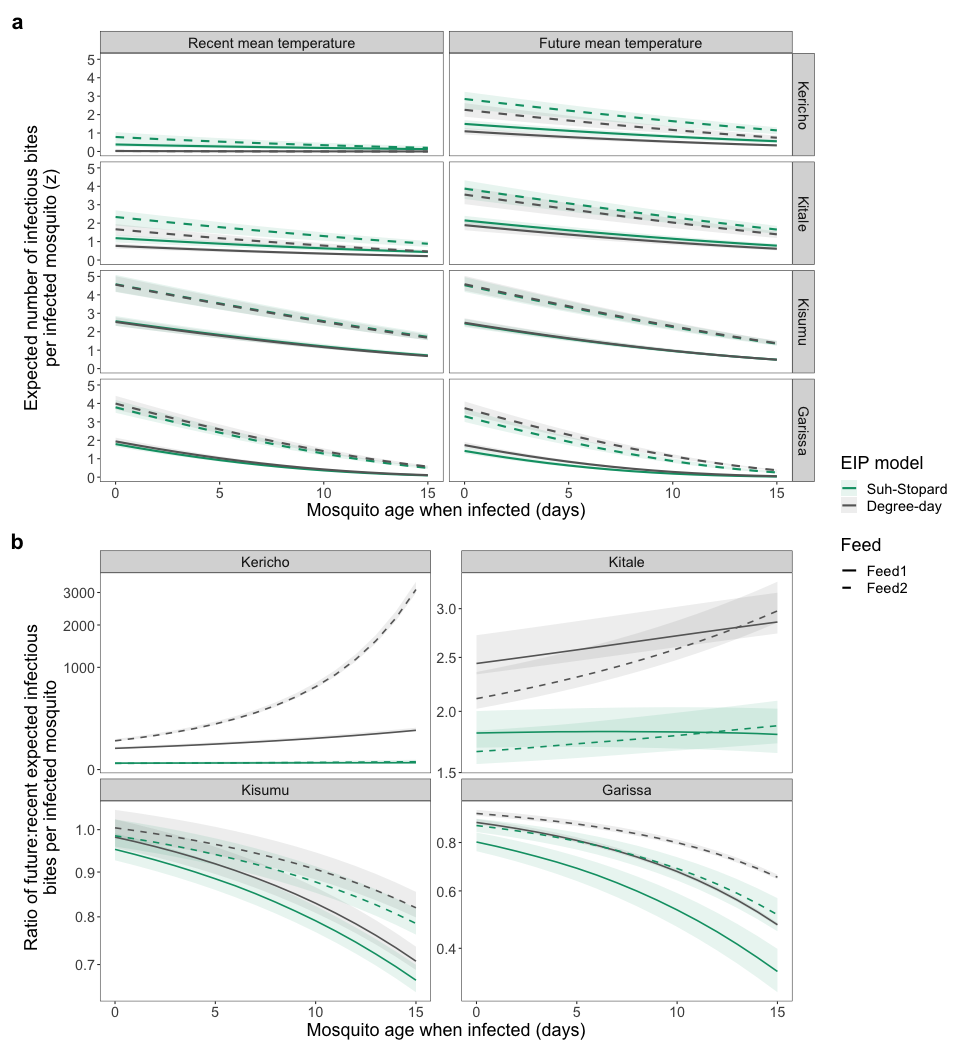


**Supplementary** **Figure 5. Changes in the expected number of infectious bites per infected mosquito (i.e. approximation of relative Vectorial Capacity, rVC) and ratio of future:recent rVC with the age at which a mosquito is infected.** (a) shows the expected number of infectious bites per infected mosquito. (b) shows the fold change in these quantities for the values estimated with future and recent mean temperatures. The lines indicate the median posterior values and uncertainty is shown by the 95% credible intervals from 100 posterior samples.


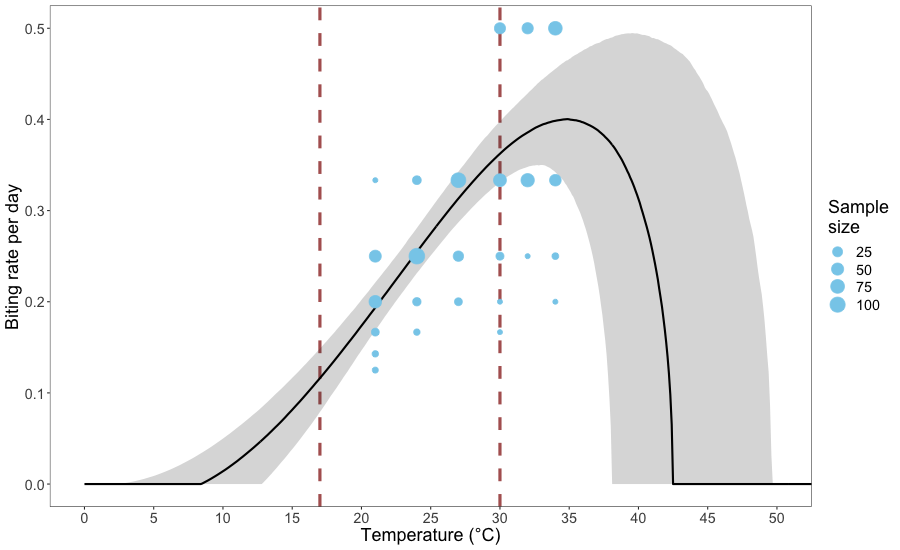


**Supplementary Figure 6. Thermal performance of biting rate of *A. stephensi.*** A Brière function is fitted on previously published biting rate data of *A. stephensi* (see methods for detail). Points show the inverse of individual mosquito gonotrophic cycle lengths. The size of the points indicates the number of gonotrophic cycles that were measured. Whether the biting rate declines at high temperatures is highly uncertain, but the temperature ranges we investigated (between the dashed red lines) are below these high temperatures. Uncertainty is shown by the 95% credible intervals.

**
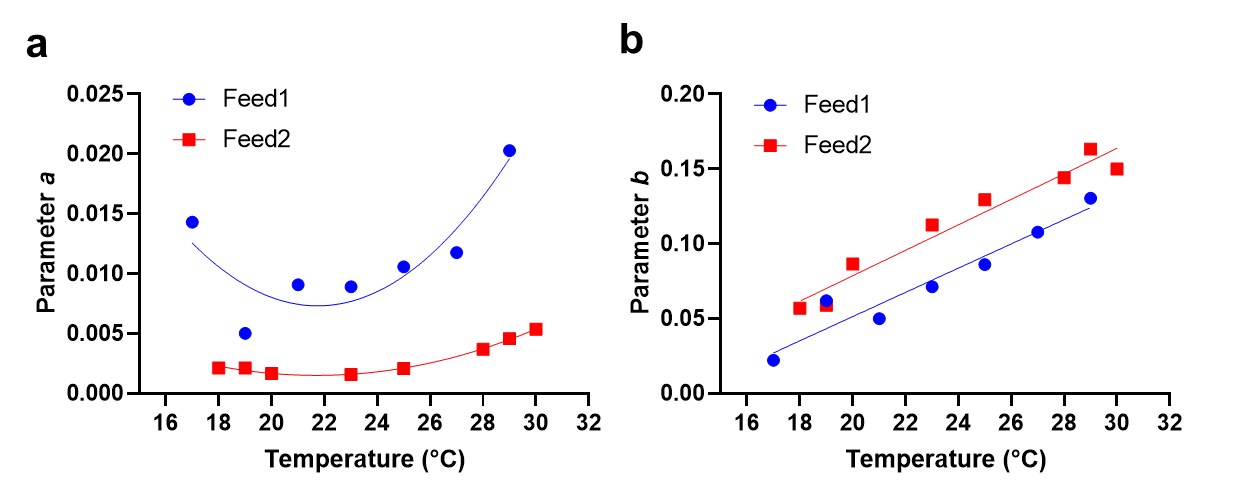
** **Supplementary Figure 7. Relationship between parameter values in Gompertz survival functions and temperature.** Values for parameter *a* and *b* determined in Gompertz functions that describe survival distribution of *A. gambiae* mosquitoes fed with parasite infected blood meals in Feed1 and Feed2 (see Supplementary Table 3 for the parameter values) are fitted with (A) quadratic and (B) linear function to describe the relationship between parameter values and temperature. Model fits and parameter values for these two functions are presented in Supplementary Table 4.

**Supplementary Table 1. Survival of *A. gambiae* mosquitoes fed with parasite infected blood meals**

| Feed | Temperature (℃) | Median Survival Days (95% CI) |
| --- | --- | --- |
| Feed1 | 17 | 38 (35 – 41) |
|  | 19 | 39 (38 – 40) |
|  | 21 | 34 (28 – 39) |
|  | 23 | 27 (25 – 30) |
|  | 25 | 22 (20 – 25) |
|  | 27 | 20 (18 – 21) |
|  | 29 | 14 (13 – 15) |
| Feed2 | 18 | 54 (53 – 56) |
|  | 19 | 52 (48 – 55) |
|  | 20 | 41 (38 – 45) |
|  | 23 | 35 (34 – 37) |
|  | 25 | 29 (28 – 32) |
|  | 28 | 24 (23 – 25) |
|  | 29 | 21 (19 – 22) |
|  | 30 | 21 (20 – 22) |

**Supplementary** **Table 2. Corrected Akaike Information Criterion (AICc) values for four different survival model fitted on cumulative survival data of malaria parasite infected *A. gambiae* mosquitoes.** Best fit models and the AICc values are bolded

| Feed | Model | AICc values for each temperature group | | | | | | | |
| --- | --- | --- | --- | --- | --- | --- | --- | --- | --- |
|  |  | 29ºC | 27ºC | 25ºC | 23ºC | 21ºC | 19ºC | 17ºC |  |
| Feed1 | **Gompertz** | **-172.9** | **-263.4** | **-312.6** | **-327.1** | **-317.2** | **-366.9** | **-328.2** |  |
|  | Weibull | -150.6 | -220.5 | -260.1 | -277.6 | -274.3 | -320.6 | -307.5 |  |
|  | Loglogistic | -133.1 | -192.4 | -226.5 | -245.6 | -247.6 | -291.0 | -285.3 |  |
|  | Negative Exponential | -106.2 | -143.1 | -167.2 | -191.4 | -215.1 | -238.7 | -299.9 |  |
|  |  | 30ºC | 29ºC | 28ºC | 25ºC | 23ºC | 20ºC | 19ºC | 18ºC |
| Feed2 | **Gompertz** | **-251.3** | **-235.5** | **-265.0** | **-311.4** | **-326.4** | **-400.0** | **-510.1** | **-520.5** |
|  | Weibull | -208.7 | -204.7 | -236.1 | -271.2 | -281.0 | -387.7 | -448.3 | -446.5 |
|  | Loglogistic | -182.6 | -182.1 | -214.2 | -240.6 | -249.9 | -355.4 | -407.8 | -404.1 |
|  | Negative Exponential | -118.3 | -112.2 | -129.0 | -136.9 | -148.5 | -187.1 | -270.0 | -280.8 |

**Supplementary Table 3. Parameter values determined by Gompertz survival model fitted on the cumulative survival data of *A. gambiae* mosquitoes fed by parasite infected blood meals**

| Feed | Parameter | Parameter values (95% CI) for each temperature group | | | | | | | |
| --- | --- | --- | --- | --- | --- | --- | --- | --- | --- |
| Feed1 |  | 29ºC | 27ºC | 25ºC | 23ºC | 21ºC | 19ºC | 17ºC |  |
|  | *a* | 0.0203 (0.0168-0.0237) | 0.0118 (0.0103-0.0132) | 0.0106 (0.0096-0.116) | 0.0089 (0.0078-0.01) | 0.0091 (0.0078-0.0103) | 0.0050 (0.0041-0.006) | 0.0143 (0.022-0.012) |  |
|  | *b* | 0.13 (0.114-0.147) | 0.108 (0.099-0.116) | 0.086 (0.07-0.092) | 0.071 (0.065-0.077) | 0.05 (0.044-0.056) | 0.062 (0.055-0.069) | 0.022 (0.015-0.029) |  |
| Feed2 |  | 30ºC | 29ºC | 28ºC | 25ºC | 23ºC | 20ºC | 19ºC | 18ºC |
|  | *a* | 0.0054 (0.0045-0.0062) | 0.0046 (0.0036-0.0055) | 0.0037 (0.0029-0.0044) | 0.0021 (0.0017-0.0024) | 0.0016 (0.0013-0.0019) | 0.0017 (0.0014-0.0019) | 0.0021 (0.0018-0.0024) | 0.0021 (0.0018-0.0024) |
|  | *b* | 0.15 (0.14-0.16) | 0.163 (0.15 – 0.176) | 0.144 (0.133-0.155) | 0.129 (0.122-0.137) | 0.113 (0.106-0.119) | 0.086 (0.082-0.091) | 0.059 (0.056-0.062) | 0.057 (0.051-0.06) |

**Supplementary Table 4. Quadratic and linear models describing the relationship between parameter values of Gompertz survival functions and temperature.** Quadratic ($G_{q}$(*T*) = *b_0_*+*b_1_T*+*b_2_T^2^*, where *T* is temperature) and linear function ($G_{l}$(*T*) = *y* + *sT*, where *T* is temperature) is fitted on values for parameter *a* and *b*, respectively. Values for parameter *a* and *b* are from previously determined Gompertz functions that describe survival distribution of *A. gambiae* mosquitoes fed with parasite infected blood meals in Feed1 and Feed2 (the parameter values presented in Supplementary Table 3). Numbers in parenthesis indicate 95% confidence intervals

| Model | Parameter/R^2^ | Feed1 | Feed2 |
| --- | --- | --- | --- |
| Quadratic | *b*_0_ | 0.118 (0.0122 – 0.223) | 0.028 (0.0229 – 0.0331) |
|  | *b*_1_ | -0.0101 (-0.0195 – -0.000781) | -0.00244 (-0.00288 – -0.00201) |
|  | *b*_2_ | 0.000233 (3.033E-5 – 0.000436) | 0.00005632 (4.721E-5 – 6.543-5) |
|  | R^2^ | 0.796 | 0.994 |
| Linear | *y* | -0.111 (-0.17 – 0.0507) | -0.0916 (-0.14 – -0.0434) |
|  | *s* | 0.00809 (0.00553 – 0.0107) | 0.00851 (0.00654 – 0.0105) |
|  | R^2^ | 0.929 | 0.949 |

**Supplementary Table 5. Experimental design for temperature treatments, dissection schedule and sample size**

| Experiment | Temperature treatment (℃) | # of container | # of mosquitoes per container | Dissection days (days post blood meal) and sample size in parentheses | |
| --- | --- | --- | --- | --- | --- |
|  |  |  |  | Midguts | Salivary glands |
| Pilot | 17 | 2 | 150 | 35, 40, 43, 47 (77) | 40, 43, 47, 50 (73) |
|  | 19 | 2 | 150 | 23 (22) | 23, 27, 30, 33, 37, 41 (123) |
|  | 21 | 2 | 150 | 17 (20) | 17, 19, 21, 23, 25, 27, 29, 33 (154) |
|  | 23 | 2 | 150 | 13 (20) | 13, 15-18, 20, 22, 26 (141) |
|  | 25 | 2 | 150 | 10 (20) | 10, 12-15, 17, 19, 22 (162) |
|  | 27 | 2 | 150 | 7 (20) | 7, 9-12, 14, 16 (141) |
|  | 29 | 2 | 150 | 6 (20) | 6, 8-11, 13, 15, 18 (161) |
| Feed1 | 17 | 3 | 200 | 34 (30) | 34, 39, 41, 43, 45, 47, 49 (195) |
|  | 19 | 3 | 200 | 23 (30) | 23, 28-34, 36 (270) |
|  | 21 | 2 | 200 | 17 (30) | 17, 19-23, 25 (210) |
|  | 23 | 2 | 200 | 13 (30) | 13-18, 20 (190) |
|  | 25 | 2 | 200 | 10 (30) | 10-15, 17 (190) |
|  | 27 | 2 | 200 | 8 (30) | 8-13, 15 (190) |
|  | 29 | 2 | 200 | 7 (30) | 7-12, 14 (190) |
| Feed2 | 18 | 6 | 200 | 26 (60) | 26, 30, 32-39, 41, 43 (720) |
|  | 19 | 4 | 200 | 21 (40) | 21, 25, 27-34, 36, 38 (480) |
|  | 20 | 2 | 200 | 19 (30) | 19, 21-26, 28, 30 (270) |
|  | 23 | 2 | 200 | 13 (30) | 13-19, 21 (240) |
|  | 25 | 2 | 200 | 10 (30) | 10-15, 17 (210) |
|  | 28 | 2 | 200 | 7 (30) | 7-12, 14 (210) |
|  | 29 | 2 | 200 | 7 (30) | 7-12, 14 (210) |
|  | 30 | 4 | 200 | 7 (60) | 7-12, 14 (420) |

**Supplementary Table 6. Multiscale stochastic model of sporogony parameters and prior choice**

| Parameter | Parameter description | Independent model prior | Pooled model prior | Prior justification | Pooled model posterior: median and 95% CI |
| --- | --- | --- | --- | --- | --- |
| $\alpha_{GO}$ | Shape parameter of gamma distributed development times from inoculation to oocyst | $\sim N(13.7, 0.1)$ | $\sim N(13.7, 0.1)$ | Strongly informative. Observations of early oocysts were not available as mosquitoes were dissected when oocysts were late stage. The rate of  transition between $G$ and $O$ was therefore unidentifiable,  so, we specified informative priors such that the modelled  oocyst prevalence peaked prior to mosquito dissection for oocysts. | 13.71 (13.51 – 13.9 95% CI) |
| $\beta_{GO}$ | Rate parameter of gamma distributed development times from inoculation to oocyst | $\sim N(5.059835, 0.1)$ | $\sim N(5.059835, 0.1)$ |  | 5.05 (4.85 – 5.25 95% CI) |
| $\alpha_{OS}$ | Shape parameter of gamma distributed development times from oocyst to sporozoite | $\sim N(17.9, 5.0)$ | NA | Weakly informative to give a mean development time from $O$ to $S$ of approximately 8.1 days. Prior means were obtained from a previously published model fit^2^. | NA |
| $\beta_{OS}$ | Rate parameter of gamma distributed development times from oocyst to sporozoite | $\sim N(2.2, 5.0)$ | NA |  | NA |
| $\mu$ | Mean oocyst load among infected mosquitoes (negative binomial distribution parameter) | $\sim lognormal(3,1)$ | NA | Weakly informative, with a mode of approximately 7.4 oocysts. | NA |
| $k$ | Dispersion of oocyst load among infected mosquitoes (negative binomial distribution parameter) | $\sim N(5.0, 4.0)$ | $\sim lognormal(1,0.75)$ | Weakly informative with a mode of 1.55. | 0.39 (0.26 – 0.53 95% CI) |
| $\delta_{O}$ | Probability of infection with oocysts at the mosquito scale | $\sim beta(2.5,2.5)$ | NA | Weakly informative with a mean of 0.5. Bounded between 0 and 1. | NA |
| $\delta_{S}$ | Probability of infection with sporozoites if infected with oocysts at the mosquito scale | $\sim beta(1.8,0.5)$ | NA | Informative. Skewed towards 1 following evidence that the presence of oocysts is a reliable predictor of whether a mosquito will develop sporozoites^3^. | NA |
| $a_{\alpha}$ | See equation 5. | NA | $\sim N(0, 5.0)$ | Weakly informative. At the mean values there is no temperature-dependency of the shape parameter $\alpha_{OS}$ and a mean value of 20. In previous work we estimated this value to be approximately 18^2^. | 0.12 (-1.9 – 2.17 95% CI) |
| $b_{\alpha}$ |  |  | $\sim N(0, 5.0)$ |  | -4.53 (-8.26 – -1.23 95% CI) |
| $c_{\alpha}$ |  |  | $\sim N(20, 4.0)$ |  | 38.87 (33.38 – 44.75 95% CI) |
| $m_{\beta}$ |  |  | $\sim N(0, 5.0)$ | Weakly informative. Assumes no temperature-dependency of the rate parameter ($\beta_{OS}$), with a mean development time from oocyst of sporozoites of 9.1 days, similar to previous work^2^. | 1.11 (0.85 – 1.39 95% CI) |
| $c_{\beta}$ |  |  | $\sim N(2.2, 4.0)$ |  | 2.35 (1.95 – 2.77 95% CI) |
| $a_{O}$ |  |  | $\sim N(0, 5.0)$ | Weakly informative, with a HMTP to the oocyst stage of 0.5 at the mean prior values. | -1.1 (-1.33 – -0.89 95% CI) |
| $b_{O}$ |  |  | $\sim N(0, 5.0)$ |  | 0.87 (0.7 – 1.04 95% CI) |
| $c_{O}$ |  |  | $\sim N(0, 4.0)$ |  | 1.45 (1.21 – 1.71 95% CI) |
| $a_{S}$ |  |  | $\sim N(0, 5.0)$ | Weakly informative, with a conversion efficient from the oocyst to sporozoite stage of 0.5 at the mean prior values. | -4.11 (-5.9 – -2.02 95% CI) |
| $b_{S}$ |  |  | $\sim N(0, 5.0)$ |  | -1.3 (-6.6 – 1.02 95% CI) |
| $c_{S}$ |  |  | $\sim N(0, 5.0)$ |  | 10.95 (7.43 – 15.88 95% CI) |
| $a_{\mu}$ |  |  | $\sim N(0, 5.0)$ | Weakly informative with no temperature-dependency and a mean oocyst number of approximately 8 at the prior mean values. | -3.36 (-4.23 – -2.62 95% CI) |
| $b_{\mu}$ |  |  | $\sim N(0, 5.0)$ |  | 2.13 (1.7 – 2.62 95% CI) |
| $c_{\mu}$ |  |  | $\sim N(10, 5.0)$ |  | 3.17 (1.98 – 4.67 95% CI) |
| $d$ |  |  | $\sim N(8, 5.0)$ |  | 21.03 (16.62 – 25.8 95% CI) |

**References**

1 Nikolaev, B. P. [On the influence of temperature on the development of malaria plasmodia in the mosquito]. *Trans. Pasteur Inst. Epi. Bact. Leningrad* **2** (1935).

2 Stopard, I. J., Churcher, T. S. & Lambert, B. Estimating the extrinsic incubation period of malaria using a mechanistic model of sporogony. *PLoS Comput Biol* **17**, e1008658 (2021). <https://doi.org/10.1371/journal.pcbi.1008658>

3 Stone, W. J. *et al.* The relevance and applicability of oocyst prevalence as a read-out for mosquito feeding assays. *Sci Rep* **3**, 3418 (2013). <https://doi.org/10.1038/srep03418>
