# Supplementary material for: Estimating the effects of temperature on transmission of the human malaria parasite, Plasmodium falciparum
Source: Nat Commun. 2024 Apr 22;15:3230. doi: 10.1038/s41467-024-47265-w (PMC11035611; doi:10.1038/s41467-024-47265-w)
Supplement: Supplementary file 3 — Reporting Summary [file 41467_2024_47265_MOESM3_ESM.pdf]

nature portfolio

Corresponding author(s): Eunho Suh

Last updated by author(s): Mar 18, 2024

Reporting Summary

Nature Portfolio wishes to improve the reproducibility of the work that we publish. This form provides structure for consistency and transparency in reporting. For further information on Nature Portfolio policies, see our Editorial Policies and the Editorial Policy Checklist.

Statistics

For all statistical analyses, confirm that the following items are present in the figure legend, table legend, main text, or Methods section.

n/a	Confirmed	
☐	☒	The exact sample size (n) for each experimental group/condition, given as a discrete number and unit of measurement
☐	☒	A statement on whether measurements were taken from distinct samples or whether the same sample was measured repeatedly
☐	☒	The statistical test(s) used AND whether they are one- or two-sided<br>*Only common tests should be described solely by name; describe more complex techniques in the Methods section.*
☒	☐	A description of all covariates tested
☒	☐	A description of any assumptions or corrections, such as tests of normality and adjustment for multiple comparisons
☐	☒	A full description of the statistical parameters including central tendency (e.g. means) or other basic estimates (e.g. regression coefficient) AND variation (e.g. standard deviation) or associated estimates of uncertainty (e.g. confidence intervals)
☐	☒	For null hypothesis testing, the test statistic (e.g. F, t, r) with confidence intervals, effect sizes, degrees of freedom and P value noted<br>*Give P values as exact values whenever suitable.*
☐	☒	For Bayesian analysis, information on the choice of priors and Markov chain Monte Carlo settings
☒	☐	For hierarchical and complex designs, identification of the appropriate level for tests and full reporting of outcomes
☒	☐	Estimates of effect sizes (e.g. Cohen's d, Pearson's r), indicating how they were calculated

Our web collection on statistics for biologists contains articles on many of the points above.

Software and code

Policy information about availability of computer code

Data collection	No software used
Data analysis	Statistical analyses were carried out using JMP 8.0 (SAS) and Prism 8.0.2 (GraphPad). Modeling analyses were carried out using R version 4.2.1 and Stan version 2.21.0. Code for the modeling study is available from https://github.com/IsaacStopard/mSOS_gambiae_only (https://doi.org/10.5281/zenodo.10688356).

For manuscripts utilizing custom algorithms or software that are central to the research but not yet described in published literature, software must be made available to editors and reviewers. We strongly encourage code deposition in a community repository (e.g. GitHub). See the Nature Portfolio guidelines for submitting code & software for further information.

Data

Policy information about availability of data

All manuscripts must include a data availability statement. This statement should provide the following information, where applicable:

- Accession codes, unique identifiers, or web links for publicly available datasets
- A description of any restrictions on data availability
- For clinical datasets or third party data, please ensure that the statement adheres to our policy

All relevant data are available from the manuscript or https://github.com/IsaacStopard/mSOS_gambiae_only (https://doi.org/10.5281/zenodo.10688356). Source data for Fig. 4 is provided with the paper.

Research involving human participants, their data, or biological material

Policy information about studies with human participants or human data. See also policy information about sex, gender (identity/presentation), and sexual orientation and race, ethnicity and racism.

Reporting on sex and gender	Not applicable (Human whole blood collected from anonymous donors was purchased from a retail supplier).
Reporting on race, ethnicity, or other socially relevant groupings	Not applicable (Human whole blood collected from anonymous donors was purchased from a retail supplier).
Population characteristics	Not applicable (Human whole blood collected from anonymous donors was purchased from a retail supplier).
Recruitment	Not applicable (Human whole blood collected from anonymous donors was purchased from a retail supplier).
Ethics oversight	Not applicable (Human whole blood collected from anonymous donors was purchased from a retail supplier).

Note that full information on the approval of the study protocol must also be provided in the manuscript.

Field-specific reporting

Please select the one below that is the best fit for your research. If you are not sure, read the appropriate sections before making your selection.

☒ Life sciences ☐ Behavioural & social sciences ☐ Ecological, evolutionary & environmental sciences

For a reference copy of the document with all sections, see nature.com/documents/nr-reporting-summary-flat.pdf

Life sciences study design

All studies must disclose on these points even when the disclosure is negative.

Sample size	A pilot test was carried out to determine the sample size of two main experiments. Sample size was adjusted depending on the treatment group (e.g. increased sample size for extreme temperature groups where low infection was observed).
Data exclusions	No data were excluded from analyses
Replication	Two separate infection experiments were performed after a pilot test. All attempts at replication were successful.
Randomization	All treatments and handling was randomized where applicable. For example, use of incubators and temperature loggers, allocation of mosquitoes to each container, blood feeding procedure (e.g. position of containers for membrane feeding), collecting and dissecting of mosquitoes, and all procedures in transmission and survival experiments were randomized.
Blinding	We do not consider blinding to be necessary in this study. Blinding is important when there are clear a priori expectations for treatment effects. In this study, we are evaluating the consequences of certain treatments but have no biases towards any particular effect sizes or direction. Moreover, dissection and scoring of mosquitoes was conducted by 4 experimenters and this should prevent bias.

Reporting for specific materials, systems and methods

We require information from authors about some types of materials, experimental systems and methods used in many studies. Here, indicate whether each material, system or method listed is relevant to your study. If you are not sure if a list item applies to your research, read the appropriate section before selecting a response.

Materials & experimental systems

n/a	Involved in the study	
☒	☐	Antibodies
☒	☐	Eukaryotic cell lines
☒	☐	Palaeontology and archaeology
☐	☒	Animals and other organisms
☒	☐	Clinical data
☒	☐	Dual use research of concern
☒	☐	Plants

Methods

n/a	Involved in the study	
☒	☐	ChIP-seq
☒	☐	Flow cytometry
☒	☐	MRI-based neuroimaging

Animals and other research organisms

Policy information about studies involving animals; ARRIVE guidelines recommended for reporting animal research, and Sex and Gender in Research

Laboratory animals	Anopheles gambiae (G3)
Wild animals	No wild animals were used in this study.
Reporting on sex	Only female mosquitoes are responsible for malaria transmission. Approximately 10,100 female mosquitoes (morphologically identified) were used for all infection and survival experiments.
Field-collected samples	No field-collected samples were used in this study.
Ethics oversight	All experiments were conducted under Penn State Institutional Biosafety Committee protocol # 48219, which covers protocols for handling mosquitoes during rearing, blood feeding and infection

Note that full information on the approval of the study protocol must also be provided in the manuscript.

Plants

Seed stocks	*Report on the source of all seed stocks or other plant material used. If applicable, state the seed stock centre and catalogue number. If plant specimens were collected from the field, describe the collection location, date and sampling procedures.*
Novel plant genotypes	*Describe the methods by which all novel plant genotypes were produced. This includes those generated by transgenic approaches, gene editing, chemical/radiation-based mutagenesis and hybridization. For transgenic lines, describe the transformation method, the number of independent lines analyzed and the generation upon which experiments were performed. For gene-edited lines, describe the editor used, the endogenous sequence targeted for editing, the targeting guide RNA sequence (if applicable) and how the editor was applied.*
Authentication	*Describe any authentication procedures for each seed stock used or novel genotype generated. Describe any experiments used to assess the effect of a mutation and, where applicable, how potential secondary effects (e.g. second site T-DNA insertions, mosiacism, off-target gene editing) were examined.*
